# Association of sleep duration with sarcopenic obesity in multi-ethnic older adults: findings from the WCHAT Study

**DOI:** 10.1186/s12877-022-03543-0

**Published:** 2022-11-24

**Authors:** Mei Yang, Yan Zhang, Wan-yu Zhao, Mei-ling Ge, Xue-lian Sun, Shu-li Jia, Bi-rong Dong

**Affiliations:** 1 National Clinical Research Center for Geriatrics, West China Hospital, Sichuan University, GuoXueXiang 37, 610041 Chengdu, China; 2 Center of Gerontology and Geriatrics, West China Hospital, Sichuan University, GuoXueXiang 37, 610041 Chengdu, China

**Keywords:** Long sleep, Sarcopenia, Obesity, Elderly

## Abstract

**Objective:**

Sarcopenic obesity is a prevalent geriatric syndrome, characterized by concurrence of sarcopenia and obesity. Sleep duration is linked to both obesity and sarcopenia. However, little was known regarding the association of sleep duration with sarcopenic obesity. In this study, we aimed to examine the association of sleep duration with sarcopenic obesity in multi-ethnic community-dwelling older adults.

**Methods:**

Sarcopenia was defined according to the criteria established by Asian Working Group for Sarcopenia (AWGS) 2019. Obesity was defined as body fat percentage above the 60th percentile specified by sex. Sarcopenic obesity was defined as concurrence of obesity and sarcopenia. Sleep duration was collected by a self-reported questionnaire and was further divided into 5 groups: “<6 h”, “6–7 h”, “7–8 h”, “8–9 h” (reference group) and “≥9 h” (long sleep). Logistic regressions were adopted to examine the association.

**Results:**

2256 multi-ethnic adults aged 60 and over from the West China Health and Aging Trend (WCHAT) study were involved for present study. Overall, 6.25% of the participants were classified as sarcopenic obesity. In the fully adjusted model, long sleep duration (≥ 9 h) was significantly associated with sarcopenic obesity compared with reference group (OR = 1.81, 95%CI = 1.10–2.98, P = 0.019). However, in subgroup analysis, this association can only be observed in male (OR 1.98, 95% CI = 1.02–3.87, P = 0.043) not in female (OR = 1.83, 95%CI = 0.85–3.94, P = 0.118). Regarding ethnic difference, Han older adults with long sleep duration (≥ 9 h) presented increased risk of sarcopenic obesity while ethnic minorities did not.

**Conclusion:**

This study disclosed that long sleep duration significantly increased the risk of sarcopenic obesity among older adults. And our findings highlight the critical role of assessing sleep duration to identify individuals at risk of sarcopenic obesity.

## Introduction

Sarcopenic obesity [1] (SO), characterized by concurrence of sarcopenia and obesity, is a health-threatening geriatric syndrome. As we age, fat gain usually occurs in conjunction with muscle loss, which potentiates the development of sarcopenic obesity. Sarcopenic obesity is prevalent in older adults and the prevalence ranges from 2.75–20% [2]. Significant variation exists in sarcopenic obesity prevalence due to different diagnostic criteria. Obesity per se could induce muscle loss and muscle function decline through insulin resistance, chronic inflammation and oxidation stress [3]. Sarcopenia may, in turn, enhance fat accumulation. Both sarcopenia and obesity are related to adverse health outcomes. However, sarcopenic obesity may augment deleterious effect of the either sarcopenia or obesity. It is reported that individuals with sarcopenic obesity had 2-fold increased risks of frailty (odds ratio (OR) = 2.0, 95% confidence interval (95% CI) = 1.42–2.82), nearly 2-fold increased risk of activities of daily living (ADL) disability (OR = 1.58, 95% CI = 1.12–2.24)[4]. Besides, sarcopenic obesity contributes to increased risks of falls (risk ratio (RR) = 1.30, 95% CI = 1.10–1.54) and fractures (incidence rate ratio (IRR) = 1.88, 95% CI = 1.09–3.23) [5]. Moreover, individual with sarcopenic obesity are more prone to cardiometabolic diseases [6] and are associated with higher risk of all-cause mortality(pooled hazards ratio (HR) = 1.21, 95% CI = 1.10–1.32) [7].

To date, specific interventions for sarcopenic obesity are lacking. Yet, potential managements for sarcopenia such as exercise (aerobic or resistance exercise), nutritional supplements (protein, vitamin D, creatine, essential amino acids, multi-nutrients and catechin tea) and testosterone [8, 9], may also be therapeutic strategies for sarcopenic obesity. Besides, a combination of nutritional supplementation and exercise is demonstrated to reduce serum myostatin level[10] and improve hand grip strength [11]. In addition, meta-analysis conducted by Hsu et al. showed that resistance exercise could decrease fat mass as well as improve grip strength. And Low-calorie high-protein diet decreased fat mass but did not affect muscle quality and quantity [12]. Although some novel therapeutic strategies such as testosterone, myostatin inhibitor, selective androgen receptor modulators, anamorelin, neuromuscular activation (whole-body vibration therapy using electric stimuli or tai chi) are under investigation [13], there is still a long way to go. Therefore, identifying risk factors for sarcopenic obesity is a priority.

A sufficient amount of sleep is crucial for maintaining the health of older adults both physically and psychologically. Too little or too much sleep have been proven to contribute to obesity [14, 15], metabolic syndrome [16], diabetes [17], cardiovascular disease [18] and mortality [19]. In addition, sarcopenia has been associated with sleep duration in several studies [20–22]. It is reported that individuals with < 6 h of sleep was associated with 2.76 times increased odds of sarcopenia, while individuals with ≥ 8 h of sleep was related to 1.89 times increased odds of sarcopenia [20] compared with individuals with 6–8 h of sleep. Considering sleep duration is connected to obesity and sarcopenia, it may also be involved in the development of sarcopenic obesity.

To our knowledge, little was known regarding the association of sleep duration with sarcopenic obesity. This study aimed to find out if sleep duration was associated with sarcopenic obesity among multi-ethnic community-dwelling older adults of western China.

## Methods

### Study population

Analysis was conducted based on data from the West China Health and Aging Trend (WCHAT) study. Details of the WCHAT study have been previously described [23]. The WCHAT study was conducted under the guidance of Declaration of Helsinki and was approved by the Ethics Committee of West China Hospital, Sichuan University (reference: 2017–445). Besides, this study registered at the Chinese Clinical Trial Registry (number ChiCTR1800018895; date of first registration 16/10/2018). Each participant signed a written informed consent prior to enrollment.

The followings were the inclusion criteria:1) Participants from the WCHAT study who aged 60 years and over; 2) no missing data for appendicular skeletal muscle index, grip strength, gait speed, body fat percentage and sleep duration.

### Assessment of sleep duration

Sleep duration was collected via a self-reported questionnaire and was further categorized as < 6 h, 6–7 h, 7–8 h, 8–9 h and ≥ 9 h (long sleep). 7 to 8 h of sleep was recommended for older adults [24] and it was therefore selected as a reference in our analysis.

### Assessment of sarcopenia, obesity and sarcopenic obesity

Sarcopenia was defined in accordance with the Asian Working Group for Sarcopenia (AWGS) 2019 [25]. Appendicular skeletal muscle index (SMI) and body fat percentage were measured with a bioimpedance analyzer (InBody 770, Biospace, Korea). The cutoffs for low SMI were 7.0 kg/m^2^ and 5.7 kg/m^2^ in male and female, respectively. Dynamometer (EH101; Camry, Zhongshan, China) was used to measure grip strength. For men, the threshold for low grip strength was 28 kg and for women, it was 18 kg. The gait speed (GS) was the primary indicator of physical function and a gait speed of less than 1.0 m/s was considered abnormal. Body fat percentages above the 60th percentile designated by sex were classified as obesity [26]. Concurrence of sarcopenia and obesity was classified as sarcopenic obesity [2].

### Covariates

Variables including age, sex, ethnicities (Han/Qiang/Tibetan/Yi/others), education level (illiteracy/primary school/secondary school and above, and marital status (married/single), smoking history, alcohol history and number of chronic diseases (0/1/≥ 2) were collected via in person interview by surveyors. The Mini Nutrition Assessment-Short Form (MNA-SF) scale was adopted to evaluate nutritional status (0 ~ 11 scores as malnutrition risk;12 ~ 14 scores as well nourished) [27]. Physical activity was assessed by using the China Leisure Time Physical Activity Questionnaire (CLTPAQ) [28]. And according to tertiles of the energy consumption, physical activity was divided into three categories: low, moderate, and high.

### Statistical analysis

Continuous data and categorical data were presented as means ± standard deviation (SD) and counts (percentages), respectively. Group differences were tested by ANOVA for continuous variables and the chi square test for categorical variables. Logistic regression models were adopted to examine the association of sleep duration with sarcopenic obesity. A multivariable model adjusted for age, sex, education, ethnicities, marital status, smoking history, alcohol history, number of chronic diseases, nutritional status and physical activity was used. Stata software, version 14.0 (Stata Corp, College Station, TX, USA), was used for the analyses. Each statistical test was two-sided and deemed statistically significant at a P < 0.05.

## Results

Overall, 2256 participants were included with a mean age of 67.6 ± 5.9 years. And 60.2% of them were female. Sarcopenic obesity accounted for 6.25% of the whole participants. Basic characteristics of the participants by sarcopenic obesity status were presented in Table 1. The sarcopenic obesity group differed significantly from the non-sarcopenic obesity group regarding age, sex, ethnicities, education level, smoking history, physical activity, nutritional status as well as sleep duration.

**Table 1:** Characteristics of participants by sarcopenic obesity status

	Total (n = 2256)	Non-sarcopenic obesity (n = 2115)	Sarcopenic obesity (n = 141)	P value^b^
Age (years)^a^	67.6 ± 5.9	67.5 ± 5.8	70.1 ± 6.9	< 0.001
Female, %	1359 (60.2)	1295 (61.2)	64 (45.4)	< 0.001
Education level, %				0.002
Illiteracy	829 (36.7)	783 (37.0)	46 (32.6)	
Primary school	869 (38.5)	826 (39.1)	43 (30.5)	
Secondary school and above	558 (24.7)	506 (23.9)	52 (36.9)	
Ethnicities, %				0.031
Han	1003 (44.5)	941 (44.5)	62 (44.0)	
Qiang	642 (28.5)	611 (28.9)	31 (22.0)	
Tibetan	459 (20.3)	417 (19.7)	42 (29.8)	
Yi	121 (5.4)	117 (5.5)	4 (2.8)	
others	31 (1.4)	29 (1.4)	2 (1.4)	
Marital status, %				0.58
Married	1801 (79.8)	1691 (80.0)	110 (78.0)	
single	455 (20.2)	424 (20.0)	31 (22.0)	
History of smoking, %	422 (18.8)	385 (18.3)	37 (26.2)	0.02
History of alcohol, %	606 (27.0)	568 (27.0)	38 (27.0)	1.00
Number of chronic diseases, %				0.83
0	1236 (54.8)	1157 (54.7)	79 (56.0)	
1	576 (25.5)	543 (25.7)	33 (23.4)	
≥ 2	444 (19.7)	415 (19.6)	29 (20.6)	
Nutritional status, %				< 0.001
Well nourished	1754 (78.1)	1639 (77.9)	115 (81.6)	
Risk of malnutrition	491 (21.9)	465 (22.1)	26 (18.4)	
Physical activity, %				0.03
Low	749 (33.3)	690 (32.7)	59 (41.8)	
Moderate	750 (33.3)	697 (33.1)	53 (37.6)	
High	750 (33.3)	721 (34.2)	29 (20.6)	
Sleep duration, %				< 0.001
< 6 h	268 (11.9)	262 (12.4)	6 (4.3)	
6-7 h	254 (11.3)	236 (11.2)	18 (12.8)	
7-8 h	491 (21.8)	464 (21.9)	27 (19.1)	
8-9 h	713 (31.6)	677 (32.0)	36 (25.5)	
≥ 9 h	530 (23.5)	476 (22.5)	54 (38.3)	

^a^ Data are presented as the mean ± standard deviation (SD).

^b^ P-value for comparison between sarcopenic obesity and non-sarcopenic obesity

Table 2 presented the results of logistical regression exploring the association of sleep duration with sarcopenic obesity. Our findings showed that sarcopenic obesity was significantly more prevalent among participants who slept for longer time (≥ 9 h) (OR = 1.94, 95%CI = 1.20–3.14, P = 0.006). After controlling for confounders, the association remained significant (OR = 1.81, 95%CI = 1.10–2.98, P = 0.019). Short sleep duration (< 6 h) seemed to exert a protective effect on sarcopenic obesity (OR = 0.39, 95%CI = 0.16–0.96, P = 0.042). However, after adjusting for covariables, the association was not significant.

**Table 2:** Associations between sleep duration and sarcopenic obesity according to unadjusted and adjusted logistic regression models

	Unadjusted model OR [95% CI]	P value	Adjusted model OR [95% CI]	P value
Sleep duration				
< 6 h	0.39 (0.16, 0.96) *	0.042	0.41 (0.16, 1.04)	0.062
6-7 h	1.31 (0.70, 2.42)	0.39	1.41 (0.74, 2.65)	0.285
7-8 h	Ref.	NA	Ref.	NA
8-9 h	0.91 (0.54, 1.52)	0.73	0.94 (0.56, 1.59)	0.840
≥ 9 h	1.94 (1.20, 3.14) *	0.006	1.81 (1.10, 2.98) *	0.019

OR: Odds Ratio; CI: Confidence Interval; Ref.: Reference; NA: Non-applicable

Adjusted model was adjusted for age, sex, education, ethnicities, marital status, smoking history, alcohol history, number of chronic diseases, nutritional status and physical activity. * P < 0.05

We further explored the sex and ethnic difference in association of sleep duration with sarcopenic obesity. Results of logistic regression with fully adjusted model were presented in Tables 3 and 4. In male, long sleep duration (≥ 9 h) was significantly correlated with sarcopenic obesity (OR = 1.98, 95% CI = 1.02–3.87, P = 0.043). Nonetheless, no significant association were observed in female (OR = 1.83, 95%CI = 0.85–3.94, P = 0.118). When assessing ethnic difference, we excluded participants in Yi and other minorities because of small sample size. Here, we found that Han participants with long sleep duration (≥ 9 h) presented increased risks of sarcopenic obesity (OR = 2.59, 95%CI = 1.13–5.97, P = 0.024). Whereas, long sleep duration and sarcopenic obesity were not significantly correlated in Qiang (OR = 1.83, 95%CI = 0.70–4.78, P = 0.215) or Tibetan participants (OR = 0.99, 95%CI = 0.39–2.48, P = 0.992).

**Table 3:** Associations between sleep duration and sarcopenic obesity stratified by sex

	Male		Female	
	Adjusted model OR [95% CI]	P value	Adjusted model OR [95% CI]	P value
Sleep duration				
< 6 h	0.32 (0.71, 1.50)	0.152	0.50 (0.15, 1.66)	0.265
6-7 h	1.28 (0.49, 3.33)	0.605	1.50 (0.62, 3.63)	0.362
7-8 h	Ref.	NA	Ref.	NA
8-9 h	1.03 (0.51, 2.08)	0.929	0.89 (0.40, 2.00)	0.796
≥ 9 h	1.98 (1.02, 3.87) *	0.043	1.83 (0.85, 3.94)	0.118

OR: Odds Ratio; CI: Confidence Interval; Ref.: Reference; NA: Non-applicable

Adjusted model was adjusted for age, education, ethnicities, marital status, smoking history, alcohol history, number of chronic diseases, nutritional status and physical activity. * P < 0.05

**Table 4:** Associations between sleep duration and sarcopenic obesity stratified by ethnicities

	Han		Qiang		Tibetan	
	OR [95% CI]	P value	OR [95% CI]	P value	OR [95% CI]	P value
Sleep duration						
< 6 h	0.65 (0.19, 2.18)	0.494	NA	NA	0.16 (0.18,1.46)	0.106
6-7 h	1.71 (0.66, 4.45)	0.268	2.03 (0.59, 6.95)	0.257	0.44 (0.10,1.85)	0.264
7-8 h	Ref.	NA	Ref.	NA	Ref.	NA
8-9 h	1.79 (0.81, 3.93)	0.145	0.25 (0.06, 1.02)	0.054	0.45 (0.15, 1.30)	0.143
≥ 9 h	2.59 (1.13, 5.97) *	0.024	1.83 (0.70, 4.78)	0.215	0.99 (0.39, 2.48)	0.992

OR: Odds Ratio; CI: Confidence Interval; Ref.: Reference; NA: Non-applicable

Adjusted model was adjusted for age, sex, education, marital status, smoking history, alcohol history, number of chronic diseases, nutritional status and physical activity. * P < 0.05

## Discussion

This study aimed to investigate whether sleep duration was related to sarcopenic obesity in multi-ethnic community-dwelling older adults of western China. The findings of our study demonstrated that long sleep duration (≥ 9 h) increased the risk of sarcopenic obesity among older adults. However, this association disappeared in female after stratification by sex. Regarding ethnic difference, the Han older adults with long sleep presented increased risks of sarcopenic obesity while the ethnic minorities did not. Our study provided a new idea for identifying individuals at high-risk of developing sarcopenic obesity.

The results of our study revealed that long sleepers (≥ 9 h) were more prone to developing sarcopenic obesity among older adults, whereas shorter sleepers were not. Presently, association of sleep duration with sarcopenic obesity has not yet been directly studied. But correlation between sleep duration and sarcopenia has already been uncovered. Previous meta-analysis including several cross-sectional analyses disclosed a U-shaped association of sleep duration with sarcopenia [29]. However, a recent longitudinal study [30] demonstrated that only long sleep duration significantly predicted the progression of sarcopenia (OR = 1.66, 95%CI = 1.02–2.69, P = 0.04), which potentially supported our findings. Sleep duration and obesity have also been associated. Meta-analysis of prospective studies conducted by Wu et al. showed that short sleep was correlated to incident obesity while long sleep was not [31]. Yet, in another meta-analysis, long sleep [32] was found to be significantly associated with obesity (OR = 1.08, 95%CI = 1.02–1.15). The inconsistent results may due to difference in measurement methods and adjustments for covariates in individual research. Despite of the discrepancy, these findings implied possible association of long sleep duration with sarcopenic obesity from clinical perspective. And our study further confirmed this association.

The biological mechanisms, which link sarcopenic obesity with long sleep duration remains obscure. However, possible mechanisms linking long sleep duration with sarcopenia or obesity may also play a role in the development of sarcopenic obesity. First, long sleep duration is reported to be associated with insulin resistance [33] and chronic inflammation [34], which are also involved in obesity and sarcopenia. Insulin resistance is inversely associated with circulating insulin-like growth factor (IGF1) [35] and it is reported that insulin resistance could inhibit the synthesis of muscle protein by downregulating the IGF-1/phosphatidylinositol 3-kinase (PI3K)/ protein kinase B (Akt) and mammalian Target of Rapamycin (mTOR) activity. Besides, insulin resistance could also promote muscle atrophy by inhibiting IGF1/PI3K/AKT pathways, activating fork head box O transcription factor (FOXO), enhancing atrogin-1 and MuscleRING-Finger-1 effects [36]. And higher level of proinflammatory markers, for example interluckin-6, may promote muscle proteolysis by upregulating ubiquitin-proteasome activity and activating the NF-kB pathways [37]. Moreover, sleep disorders could reduce the secretions of testosterone, which could upregulate the expression of myostatin and Regulated in Development and DNA Damage responses 1 (REDD1), promoting protein proteolysis [36].

On the other hand, long sleep duration is associated with decreased physical activity, a contributing factor for both sarcopenia [38] and obesity [39]. Present studies revealed that self-reported longer sleep (> 9 h) was related to lower daily physical activity. And physical activity decreased by 29 min per additional sleep hour [40–42]. Therefore, it may act as a mediator between long sleep and sarcopenic obesity. It is postulated that excessive body fat and low muscle mass/function resulting from low physical activity may in turn disrupt normal sleep pattern via systematic inflammation, insulin resistance and some toxic brain metabolites which could not be timely removed [43], forming a vicious cycle. Moreover, the molecular clock and circadian rhythms are essential for maintaining and adapting skeletal muscle [44] as well as regulating lipid metabolism [45]. Physical activity can modulate the molecular clock in skeletal muscle, affecting phase of circadian rhythms [46]. And exercise has been considered as an effective intervention for sarcopenia [47] and metabolic disease by re-setting circadian clock [48].

Our study firstly identified the association of long sleep duration with sarcopenic obesity in multi-ethnic community-dwelling older adults and explored sex and ethnic difference in their associations. There nevertheless existed some limitations. First, findings of this cross-sectional study failed to reveal a causal link between sarcopenic obesity and sleep duration. And these findings need to be confirmed through longitudinal research. Secondly, data on sleep duration was self-reported, which may introduce bias. Thirdly, we may not able to directly generalize our results to other regions of China. Finally, there were other potential con-founders we failed to address such as dietary preference, detailed diseases and possible drug use.

## Conclusion

This study disclosed that long sleep duration significantly increased the risk of sarcopenic obesity among older adults. And our findings highlight the critical role of assessing sleep duration to identify individuals at risk of sarcopenic obesity.

## Data Availability

The data that support the findings of this study are available from National Clinical Research Center for Geriatrics, West China Hospital but restrictions apply to the availability of these data, which were used under license for the current study, and so are not publicly available. Data are however available from the corresponding author upon reasonable request and with permission of National Clinical Research Center for Geriatrics, West China Hospital.

## Abbreviations

SO: Sarcopenic obesity
OR: Odds ratio
CI: Confidence interval
ADL: Activities of daily living
RR: Risk ratio
IRR: Incidence rate ratio
HR: Hazards ratio
AWGS: Asian Working Group for Sarcopenia
SMI: Appendicular skeletal muscle index
GS: Gait speed
MNA-SF: The Mini Nutrition Assessment-Short Form
CLTPAQ: The China Leisure Time Physical Activity Questionnaire
SD: Standard deviation
IGF1: Insulin-like growth factor
PI3K: Phosphatidylinositol 3-kinase
Akt: Protein kinase B
mTOR: Mammalian Target of Rapamycin
FOXO: Fork head box O transcription factor
REDD1: Regulated in Development and DNA Damage responses 1
